# Left atrial appendage closure in nonvalvular atrial fibrillation patients with percutaneous coronary intervention

**DOI:** 10.1186/s12872-022-02865-6

**Published:** 2022-10-03

**Authors:** Yunan Yu, Jing Xu, Liang Wang, Zi Ye, Zhisong Chen, Fadong Chen

**Affiliations:** 1 Department of Cardiology, Tongji Hospital, Tongji University School of Medicine, Shanghai, China; 2 Department of Cardiology, Shanghai East Hospital, Tongji University School of Medicine, Shanghai, China

**Keywords:** Left atrial appendage closure, Atrial fibrillation, Percutaneous coronary intervention, Thrombosis, Bleeding

## Abstract

**Objectives:**

Nonvalvular atrial fibrillation (NVAF) concomitant with coronary artery disease (CAD) may increase the risk of thromboembolism. Antithrombotic therapy for NVAF patients with percutaneous coronary intervention (PCI) remains contradictory and challenging. This study aimed to assess the safety and efficacy of left atrial appendage closure (LAAC) in a cohort of patients with NVAF and PCI.

**Methods:**

A total of 109 patients undergoing LAAC procedures between March 2017 and December 2020 were categorized into 2 groups, Group I included 36 patients with PCI while group II included 73 patients without. Peri-procedural and long-term complications, as well as ischemia and bleeding events, were retrospectively analyzed.

**Results:**

Group I had more diabetes mellitus (55.6% vs. 26.0%; p = 0.003), higher CHA2DS2-VASc scores (5.44 ± 1.85 vs. 4.22 ± 1.64; p = 0.002) and HAS-BLED scores (3.39 ± 0.93 vs. 2.74 ± 1.05; p = 0.003) compared to Group II. Procedure-related complications within 7 days were similar in both groups (8.3% vs. 8.2%; P = 1.000). Over a median follow-up period of 20.9 months, there were no significant differences between two subgroups with regard to cardiovascular death (2.8% vs. 0%, p = 0.330), stroke/transient ischemic attack (2.8% vs. 5.5%, p = 1.000), major bleeding (0% vs. 2.7%, p = 1.000) and device-related thrombus (8.3% vs. 1.4%, p = 0.104). The observed annualized thromboembolic and major bleeding events determined by Kaplan–Meier analysis decreased by 82.4% and 100% in group I, 55.9% and 75.8% in group II, respectively.

**Conclusion:**

LAAC is a safe and effective option for stroke prevention in NVAF patients with PCI.

## Introduction

Atrial fibrillation (AF) is the most common cardiac arrhythmia in clinical practice associated with a significantly increased risk of embolic stroke [1]. Oral anticoagulation (OAC) is recommended for stroke prophylaxis in patients with nonvalvular AF (NVAF), but there are still several limitations and side effects in the clinical setting [2–4]. Recently, percutaneous left atrial appendage closure (LAAC) has developed as an important therapeutic option for NVAF patients with high thromboembolic risk or relative/absolute contraindications to long-term OAC [5].

It is well known that a high incidence and prevalence of coronary artery disease (CAD) occurred in patients with AF [6–8]. AF patients with percutaneous coronary intervention (PCI) always carry high ischemic risk because they have more comorbidities such as diabetes, hypertension, renal insufficiency, and peripheral arterial disease. The optimal antithrombotic regimen remains challenging and needs tailored treatment for these individuals [9]. The combination of dual antiplatelet therapy and oral anticoagulants is associated with high risk of major bleeding, and the optimal use of triple therapy in clinical settings remains controversial.

Currently, LAAC is performed widely for antithrombotic events prevention in NAVF patients with heart failure, chronic kidney disease, and high bleeding risk [10–12]. Moreover, in NVAF patients with PCI who may require both OAC and antiplatelet therapy, LAAC may have the potential benefit of reducing the usage of OAC. Whether LAAC is the optimal choice for patients with PCI remains unknown.

In this study, we aimed to evaluate the long-term safety and efficacy of LAAC in NVAF patients with PCI.

## Methods

### Patients

A total of 109 consecutive NVAF patients undergoing successful LAAC between March 2017 to December 2020 in two centers (Shanghai Tongji Hospital and Shanghai East Hospital, Tongji University, Shanghai, China) were enrolled. The indication of LAAC procedure was based on European Heart Rhythm Association/European Association of Percutaneous Cardiovascular Interventions (EHRA/EAPCI) expert consensus statement [13]. The cohort was divided into two groups, patients with PCI (Group I) and those without (Group II). All patients in Group I were diagnosed with chronic coronary syndrome. Transesophageal echocardiography (TEE) or computed tomography angiography (CTA) was performed before LAAC to confirm anatomical characteristics of left atrial appendage (LAA) and rule out LAA thrombus.

### LAAC procedure

LAAC was performed under general anesthesia with the guidance of TEE and fluoroscopy. After transseptal puncture and introduction of the delivery system to LAA, intravenous heparin was administered according to the patient’s body weight (100 IU/kg) to maintain an activated clotting time (ACT) at 250–350 s during the procedure. TEE and LAA angiogram were performed to determine the optimal device size and confirm that the device position as well as LAA sealing after the device (either LAmbre™, Lifetech Scientific Corp., Shenzhen, China; Watchman™, BostonScientific, Marlborough, MA, USA or Leftear™, Guangdong Pulse Medical Technology Co., Ltd. Zhuhai, China) was deployed. The implant success was defined as LAA closure with peri-device leak (PDL) < 5 mm under TEE imaging.

### Antithrombotic therapy at discharge and follow-up

Postimplant antithrombotic regimen in group I, including triple therapy, the combination of OAC and dual antiplatelet therapy (DAPT), dual therapy, the combination of OAC and single antiplatelet therapy (SAPT) or DAPT, and single OAC, was prescribed according to the interval time between the last PCI and LAAC. In group II, postimplant antithrombotic regimen was based on current guidelines, including warfarin (target international normalized ratio, 2.0–3.0) for 6 weeks, followed by clopidogrel and aspirin for 6 months, and aspirin alone subsequently. Adjustments in antithrombotic therapy during follow-up were based on the physician’s clinical judgment.

TEE or CTA was scheduled at 3 and 6 months post-procedure to discover any peri-device leak or device-related thrombus (DRT). Long-term follow-up was carried out by outpatient visits or telephone interviews to assess survival and complications.

### Statistical analysis

Continuous data were described as mean ± standard deviation (SD) and compared by Mann-Whitney U-test or Student’s T-test. Categorical data are described as numbers and percentages and compared by Fisher’s exact test or Chi-square test. Rates of ischemic stroke/transient ischemic attacks (TIA)/peripheral emboli and major bleeding events were calculated as the number of events per 100 patients-year. Predicted risk of annual ischemic stroke/TIA/peripheral emboli and major bleeding events were extrapolated from each patient based on CHA_2_DS_2_-VASc and HAS-BLED scores from published risk score literature [14, 15]. P-value < 0.05 was considered significant. SPSS, version 22.0 software was used to manage the data.

## Results

### Baseline characteristics

The 109 NVAF patients undergoing successful LAAC were enrolled and divided into 2 groups, Group I included 36 patients with PCI while group II included 73 patients without. Baseline characteristics of the study population were presented in (Table 1). Both CHA_2_DS_2_-VASc and HAS-BLED scores in group I were higher than those in group II (5.44 ± 1.85 vs. 4.22 ± 1.64, p = 0.002 and 3.39 ± 0.93 vs. 2.74 ± 1.05, p = 0.003 respectively). Diabetes was more prevalent (55.6% vs. 26.0%, P = 0.003) in group I. There were no statistical differences in terms of hypertension, chronic heart failure, previous stroke/TIA, and major bleedings between the two groups. AF pattern, left atrium dimension, left ventricular end-diastolic dimension, and left ventricular ejection fraction (LVEF) did not differ as well.

**Table 1:** Baseline characteristics

	Group I (n = 36)	Group II (n = 73)	P value
Male sex, *n* (%)	19 (52.8%)	39 (53.4%)	0.949
Age, years (mean ± SD)	74.31 ± 8.21	71.82 ± 6.82	0.104
Hypertension, *n* (%)	31 (86.1%)	59 (80.8%)	0.494
Diabetes mellitus, n (%)	20 (55.6%)	19 (26.0%)	0.003
Congenital heart disease, n(%)	1 (2.8%)	2 (2.7%)	1.000
Previous TIA/stroke, *n* (%)	12 (33.3%)	24 (32.9%)	0.962
Previous major bleeding, *n* (%)	4 (11.1%)	19 (26.0%)	0.073
Previous cardiac thrombus, *n* (%)	0 (0%)	6 (8.2%)	0.077
LAD, mm (mean ± SD)	46.53 ± 5.93	44.73 ± 5.53	0.197
LVEDd, mm (mean ± SD)	47.64 ± 5.16	46.79 ± 5.77	0.297
LVEF, % (mean ± SD)	61.75 ± 7.20	61.63 ± 7.37	0.856
CHA_2_DS_2_-VASc score (mean ± SD)	5.44 ± 1.85	4.22 ± 1.64	0.002
HAS-BLED score (mean ± SD)	3.39 ± 0.93	2.74 ± 1.05	0.003
Blood stasis in LAA, n (%)	0 (0%)	1 (1.4%)	1.000
Atrial fibrillation pattern			
Recent-onset	8 (22.2%)	13 (17.8%)	0.583
Paroxysmal, *n* (%)	14 (38.9%)	24 (32.9%)	0.536
Persistent or permanent, *n* (%)	22 (61.1%)	49(67.1%)	0.536
Last PCI and LAAC interval time		-	-
<7 days	2 (5.5%)		
7days-3months	4 (11.1%)		
3months-12months	6 (16.7%)		
>12months	24 (66.7%)		

Abbreviations: TIA: transient ischemic attack; LAD: left atrial diameter; LVEDd: left ventricular end-diastolic dimension; LVEF: left ventricular ejection fraction; LAA: left atrial appendage; PCI: percutaneous coronary intervention; LAAC: left atrial appendage closure.

### Procedural data and peri‑procedural complications

Successful implant was achieved in 109 (100%) patients. Procedural parameters were detailed in (Table 2).

In group I, 2 patients suffered pericardial effusions which were managed conservatively within a few days post-procedure and 1 patient suffered hypotension due to vasovagal reactions post-procedure and was recovered by treatment of rehydration. In group II, 1 severe vascular complication and 1 cardiac tamponade requiring pericardiocentesis were documented a few hours after LAAC. There were no significant differences in death, stroke, bleeding, DRT, or pericardial effusion occurrence between the two groups (Table 2).

**Table 2:** Procedural characteristics of LAAC and Peri-procedural complications

	Group I (n = 36)	Group II (n = 73)	P value
Successful implantation, n (%)	36 (100.0%)	73 (100.0%)	1.000
TEE measure			
LAA ostium width, mm (mean ± SD)	22.08 ± 3.80	22.16 ± 3.76	0.915
LAA length, mm (mean ± SD)	27. 03 ± 4.66	25.67 ± 4.49	0.136
DSA measure			
LAA ostium width, mm (mean ± SD)	21.89 ± 4.49	22.49 ± 4.10	0.642
LAA length, mm (mean ± SD)	24.86 ± 4.77	25.34 ± 4.85	0.502
Type of LAAC device, each n (%)			
Watchman™	33 (91.7%)	68 (93.2%)	1.000
LAmbre™	1 (2.8%)	4 (5.4%)	0.883
Leftear™	2 (5.5%)	1 (1.4%)	0.526
No peri-device leak, n (%)	34 (94.4%)	61 (83.6%)	0.196
Peri-device leak < 5 mm, n (%)	2 (5.6%)	12 (16.4%)	0.196
Peri-device leak ≥ 5 mm, n (%)	0 (0%)	0 (0%)	1.000
Pericardial effusion (< 3 mm), n (%)	2 (5.6%)	4 (5.5%)	1.000
ACT, s (mean ± SD)	269.18 ± 37.53	256.51 ± 43.06	0.087
Number of implantation attempts (mean ± SD)	1.19 ± 0.52	1.34 ± 0.77	0.223
Procedure Time, min (mean ± SD)	62.25 ± 23.16	69.59 ± 33.22	0.450
Peri-procedural complications			
Death, n (%)	0 (0%)	0 (0%)	1.000
Stroke, n (%)	0 (0%)	0 (0%)	1.000
Major Bleeding, n (%)	0 (0%)	0 (0%)	1.000
DRT, n (%)	0 (0%)	0 (0%)	1.000
Cardiac tamponade, n (%)	0 (0%)	1 (1.4%)	1.000
Pericardial effusion with conservative treatment, n (%)	2 (5.6%)	4 (5.5%)	1.000
Severe vascular complication, n (%)	0 (0%)	1 (1.4%)	1.000
Hypotension, n (%)	1 (2.8%)	0 (0%)	0.327

Abbreviations: TEE: transesophageal echocardiography; LAA: left atrial appendage; DSA: digital subtraction angiography; LAAC: left atrial appendage closure; ACT: activated clotting time; DRT: device-related thrombus.

### Antithrombotic medications upon discharge

**Fig. 1:**
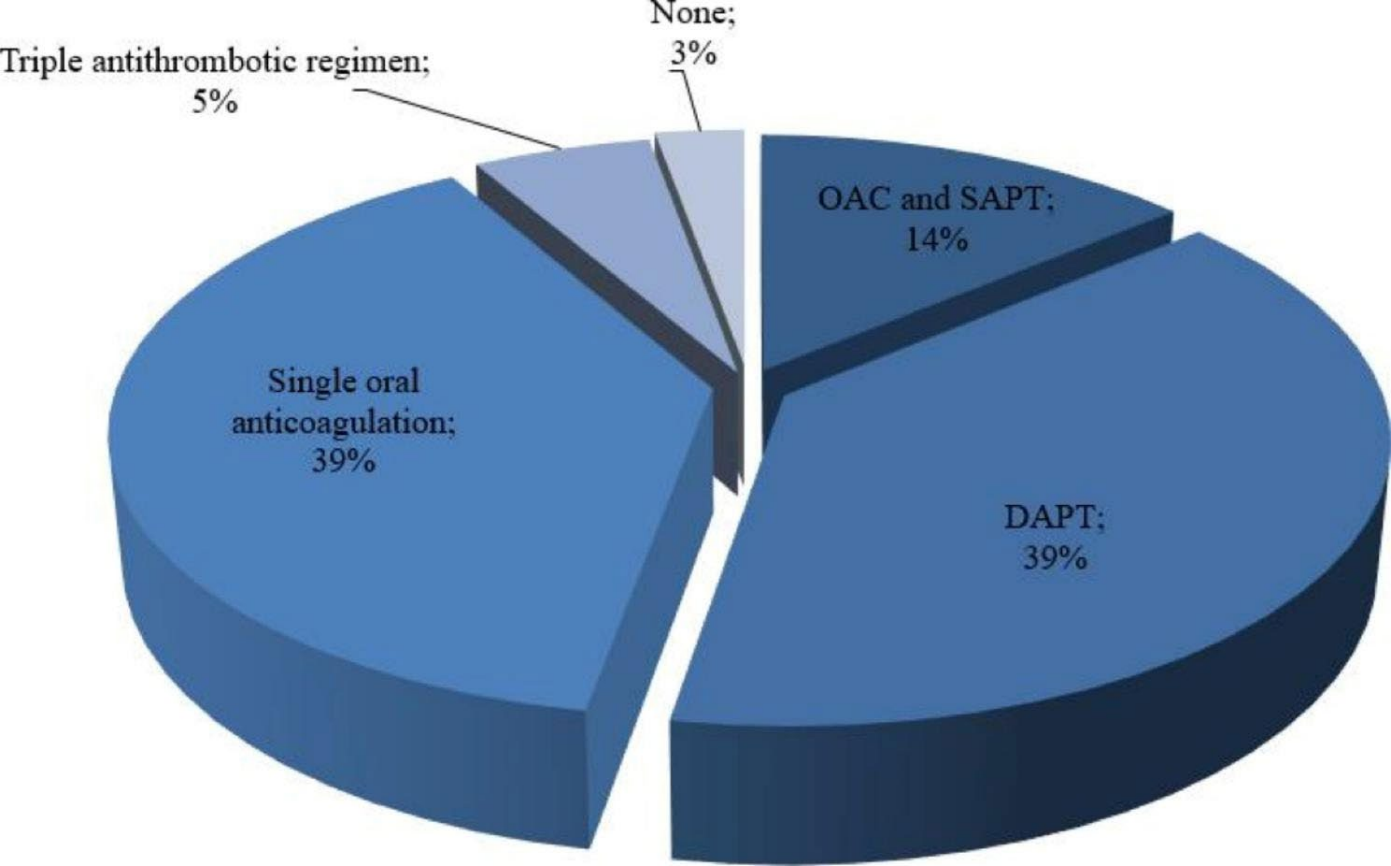
Post-procedural antithrombotic regimen of group I. DAPT: dual antiplatelet therapy; OAC: oral anticoagulation; SAPT: single antiplatelet therapy

In group I, 5 patients (13.9%) were discharged with OAC and SAPT, 14 patients (38.9%) with DAPT, 14 patients (38.9%) with single OAC. 2 patients (5.6%) were on triple antithrombotic regimen and 1 patient (2.8%) was discharged without any antithrombotic therapy (Fig. 1).

In group II, the majority of patients (61/73, 83.6%) were prescribed with single OAC, 9 patients (12.3%) with DAPT and 2 patients (2.7%) with SAPT. Only 1 patient (1.4%) had no antithrombotic therapy.

### Follow-up results

The mean follow-up period was 20.9 ± 8.63 months. Only 1 cardiovascular death (2.8%) was reported in group I at 1-month post-procedure, which was caused by complications after transcatheter aortic valve implantation (TAVI) procedure. A total of 4 (3.7%) patients were found DRT. In group I, 2 DRT patients had a history of in-stent restenosis (ISR) and were treated with anticoagulant until thrombus resolution on TEE. There were no significant differences involving DRT, heart failure, stroke/TIA, death, bleeding, cancer, PDL, and system embolism between the two groups (Table 3).

**Table 3:** Follow-up outcomes

	Group I (n = 36)	Group II (n = 73)	P value
Mean follow-up Period, months	16.7 ± 9.8	23.0 ± 7.2	0.000
All-cause death, n (%)	1 (2.8%)	2 (2.7%)	1.000
Cardiovascular death, n (%)	1 (2.8%)	0 (0%)	0.330
Non-cardiovascular death, n (%)	0 (0%)	2 (2.7%)	1.000
Stroke/TIA, n (%)	1 (2.8%)	4 (5.5%)	1.000
System embolism, n (%)	0 (0%)	1 (1.4%)	1.000
All-bleeding, n (%)	2 (5.6%)	3 (4.1%)	1.000
Major bleeding, n (%)	0 (0%)	2 (2.7%)	1.000
Gastrointestinal bleeding, n (%)	0 (0%)	2 (2.7%)	1.000
Cerebral hemorrhage, n (%)	1 (2.8%)	0 (0%)	0.330
Other bleeding, n (%)	1 (2.8%)	1 (1.4%)	1.000
Device thrombus, n (%)	3 (8.3%)	1 (1.4%)	0.104
Heart failure, n (%)	2 (5.6%)	6 (8.2%)	1.000
Cancer, n (%)	2 (5.6%)	3 (4.1%)	1.000
Peri-device leak (> 5 mm), n (%)	0 (0%)	0 (0%)	1.000

Abbreviations: TIA: transient ischemic attack.

### Efficacy for prevention of thromboembolic and hemorrhagic events

**Fig. 2:**
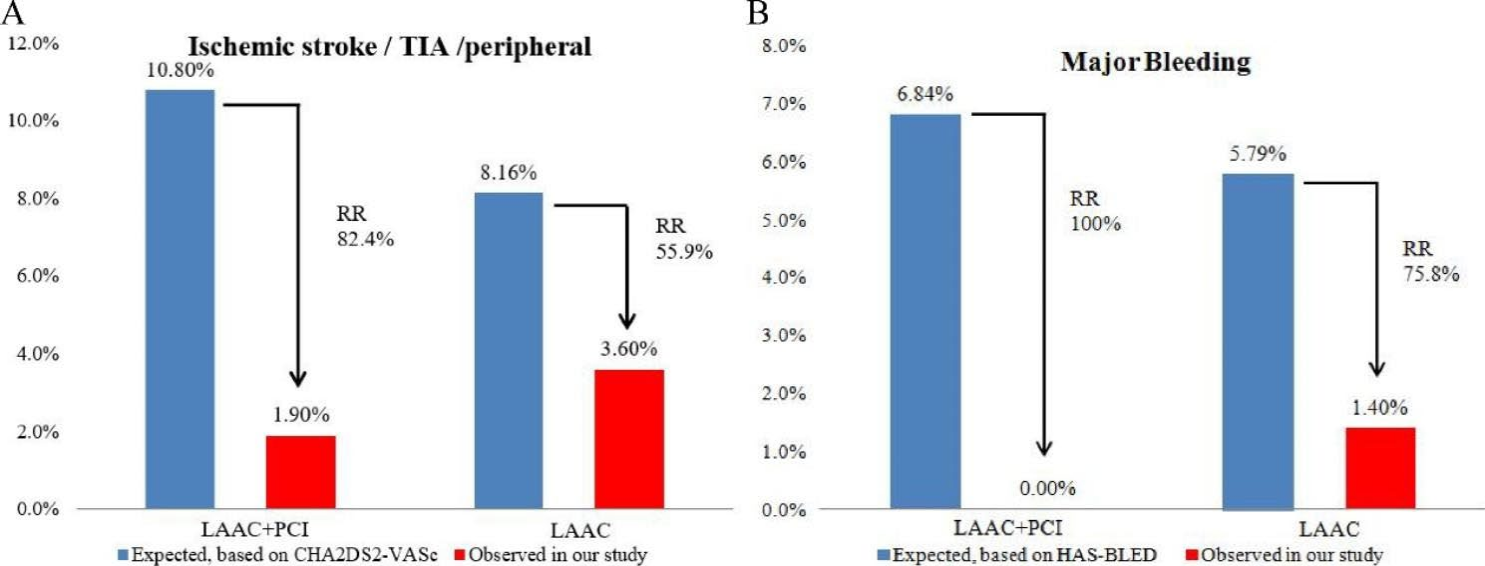
Predicted and observed rates of thromboembolic and hemorrhagic events. (A). Observed rate of thromboembolic events vs. the expected rate based on the CHA2DS2-VASc score. (B). Observed rate of major bleeding events vs. the expected rate based on the HAS-BLED score. RR: relative risk

According to Kaplan-Meier estimation, the observed annualized thromboembolic events including ischemic stroke, TIA, and systemic embolism were decreased by 82.4% in group I and 55.9% in group II respectively, compared to the predicted value (10.8% vs. 8.16%) based on CHA2DS2-VASc score (Fig. 2** A**). Meanwhile, compared to the predicted bleeding rate (6.84% vs. 5.79%) based on HAS-BLED score, the observed annualized bleeding rate was reduced by 100% and 75.8% in group I and group II respectively **(Fig. 2B)**.

## Discussion

The results of this study have shown that LAAC procedure in AF patients with PCI reduced the risk of thromboembolic events and severe bleeding over a long-term period. Percutaneous LAAC could be a safe and effective option for stroke prevention in this specific population.

The AF patients with PCI carry a high ischemic risk, therefore, short-term OAC with DAPT is recommended after PCI. The increased risk of bleeding involved with triple therapy is deemed to outweigh the benefits of thromboembolic risk reduction [16, 17]. Therefore, during the early period after PCI, when both bleeding and ischemic rates are relatively high, LAAC is expected to be useful in these particular patients. In our study, 6(16.6%) patients were performed LAAC within 3 months after PCI and no adverse cardiac events occurred. Overall, LAAC was successful in all enrolled patients (100%), consistent with 98.3% of National Cardiovascular Data Registry LAAC Registry [18]. There were no statistical differences between the two subgroups in terms of procedure-related complications and long-term outcomes over a 24-months follow-up period, suggesting LAAC appeared to be a viable option for antithrombotic prevention in NVAF patients with PCI.

The main concern is the incidence of thromboembolic and bleeding events in a population of AF with PCI. As expected, our study population had a mean CHA_2_DS_2_-VASc score of 5.44 ± 1.85 and HAS-BLED score of 3.39 ± 0.93. We recorded 1 patient in group I but 4 patients in group 2 suffered strokes/TIA. The observed results of both groups were further compared with expected rates of annual thromboembolic events (ischemic stroke, TIA, and peripheral thromboembolism) based on CHA_2_DS_2_-VASc score [14], revealing that the risk of thromboembolic events in group I decreased much more dramatically than that in group II (82.4% vs. 55.9%). It is likely explained that NVAF patients with PCI received either inadequate antithrombotic or no anticoagulation therapy due to the high risk of major bleeding and LAAC can prevent even > 99% thrombi from LAA [19]. Additionally, a majority of patients in group I were prescribed with OAC and antiplatelet drugs, particularly in patients who underwent PCI within 1 year, leading to a reduction of coronary and peripheral arterial thrombosis. Bleeding risk remains a clinical concern in NVAF patients receiving OAC and antiplatelet therapy and tailored antithrombotic regimen for individuals is always challenging. In group I, there was no major bleeding except 1 hemorrhagic stroke documented at a 24-months follow-up due to trauma. The actual annualized major bleedings risk of 0% compared favorably to an estimated 6.84% based on HAS-BLED score [15], with a dramatic reduction of 100%. LAAC offers an alternative mechanical approach for NVAF patients with PCI because it allows OAC discontinuation and consequently, leads to a reduced risk of bleeding.

There has been increasing concern about DRT during follow-up after LAAC [20–22]. The rate of DRT (3.6%) in this study was comparable to other published data of 4.1% [23] and 3.3% [24]. In group I, 3 patients were found late DRT and 2 of them had a reduced LVEF which was a proven factor associated with DRT formation after LAAC [22]. They were all dissolved with intense anticoagulant treatment without ischemic stroke. Interestingly, in group I, 2 DRT patients had prior ISR. Both ISR and DRT were partly due to poor device endothelialization as a consequence of endothelial dysfunction [25, 26], indicating ISR may hold a potential role in DRT after LAAC. Moreover, these findings support the notion that for the patients undergoing LAAC with ISR history, close follow-up with TEE or CTA and intensive antithrombotic therapy are crucial for DRT prevention.

Our study has important clinical relevance. Drug management of NVAF patients with PCI is complex and challenging because the individualized antithrombotic strategy should be made according to guidelines based on ischemic and bleeding risk [27, 28]. DAPT therapy after LAAC, which is consistent with the drug regimen after PCI, has been recently proven to be feasible and safe [29, 30]. In our study, we found that LAAC is efficient and safe in NVAF patients with PCI, and therefore may be an ideal choice to prevent stroke and other thrombotic complications in this specific high-risk group.

## Limitations

The current study has several limitations. First, the sample size was relatively small and it was not a randomized trial. Further larger prospective and randomized controlled studies are needed to confirm the conclusion. Second, considering the relatively small sample size, we did not analyze the differences by the time from the previous PCI which could be extremely meaningful. In the future, by increasing the sample size and modifying entry criteria, we will further study the safety and efficacy of LAAC in AF patients with PCI. Furthermore, the last follow-up using phone contact was prone to subjective bias which might have an impact on the outcome of our study.

## Conclusion

LAAC appears to be safe and feasible for NVAF patients with PCI carrying high ischemic and bleeding risk. This observation may provide novel evidence of LAAC application in clinical practice.

## Data Availability

The datasets used and/or analyzed during the current study are available from the corresponding author (chenfadong0819@163.com).

## Abbreviations

NVAF: Nonvalvular atrial fibrillation
CAD: Coronary artery disease
PCI: Percutaneous coronary intervention
LAAC: Atrial fibrillation
OAC: Oral anticoagulation
EHRA/EAPCI: European Heart Rhythm Association/European Association of Percutaneous Cardiovascular Interventions
TEE: Transesophageal echocardiography
CTA: Computed tomography angiography
DAPT: Dual antiplatelet therapy
SAPT: Single antiplatelet therapy
LAA: Left atrial appendage
ACT: Activated clotting time
PDL: Peri-device leak
DRT: Device-related thrombus
SD: Standard deviation
TIA: Transient ischemic attacks
LVEF: Left ventricular ejection fraction
TAVI: Transcatheter aortic valve implantation
ISR: In-stent restenosis.
